# Osmic Acid in Neuralgia

**Published:** 1898-04-16

**Authors:** 


### OSMIC ACID IN NEURALGIA.

At the last meeting of the Medical Society, Mr. G. R. Turner showed a female patient, aged 33, who had suffered for two years from neuralgia which had resisted all treatment. The pain originally involved the infraorbital nerve, but had extended to other divisions of the fifth, and had been accompanied by a discharge from the right nostril. Neither the antrum nor the nasal fossæ showed any abnormality on ordinary examination. Before exploring the antrum, as a preliminary if necessary to the removal of the Gasserian ganglion, 15 minims of a 1 per cent. solution of osmic acid were injected, by an ordinary hypodermic syringe, into the infraorbital canal. A considerable amount of pain and tenderness resulted, but when this had passed away, *i.e.*, in about 10 days, the pain did not return, and the patient had remained free from her old trouble for some two months. Whether the relief was due to the well-known but more or less transient effects of traumatism, or to the destructive influence of osmic acid on nerve fibres, is a question which time may perhaps decide. This appears to be the first case in which this treatment has been adopted in England, but it has been advised by Neuber, Eulenberg, and Franck.

